# Is There a Relationship between Voice Quality and Obstructive Sleep Apnea Severity and Cumulative Percentage of Time Spent at Saturations below Ninety Percent: Voice Analysis in Obstructive Sleep Apnea Patients

**DOI:** 10.3390/medicina58101336

**Published:** 2022-09-23

**Authors:** Serhat Yaslıkaya, Ayşegül Altıntop Geçkil, Zehra Birişik

**Affiliations:** 1 Department of Otorhinolaryngology, Faculty of Medicine, Adıyaman University, Adıyaman 02100, Turkey; 2 Department of Chest Diseases, Faculty of Medicine, Malatya Turgut Özal University, Malatya 44210, Turkey; 3 Department of Speech and Language Therapy, Malatya Training and Research Hospital, Malatya 44000, Turkey

**Keywords:** Apnea, inflammation, polysomnography, phonation

## Abstract

*Background and Objectives*: Apnea hypopnea index is the most important criterion in determining the severity of obstructive sleep apnea (OSA), while the percentage of the total number of times which oxygen saturation is measured below 90% during polysomnography (CT90%) is important in determining the severity of hypoxemia. As hypoxemia increases, inflammation will also increase in OSA. Inflammation in the respiratory tract may affect phonation. We aimed to determine the effects of the degree of OSA and CT90% on phonation. *Materials and Methods*: The patients were between the ages of 18–60 years and were divided into four groups: normal, mild, moderate, and severe OSA. Patients were asked to say the vowels /α:/ and /i:/ for 5 s for voice recording. Maximum phonation time (MPT) was recorded. Using the Praat voice analysis program, Jitter%, Shimmer%, harmonics-to-noise ratio (HNR), and f0 values were obtained. *Results*: Seventy-two patients were included. Vowel sound /α:/; there was a significant difference for Jitter%, Shimmer%, and HNR measurements between the 1st and the 4th group (*p* < 0.001, *p* < 0.001, and *p* < 0.001, respectively) and a correlation between CT90% and Shimmer% and HNR values (*p* < 0.001 and *p* < 0.021, respectively). Vowel sound /i:/; there was a significant difference in f0 values between the 1st group and 2nd and 4th groups (*p* < 0.028 and *p* < 0.015, respectively), and for Jitter%, Shimmer%, and HNR measurements between the 1st and 4th group (*p* < 0.04, *p* < 0.000, and *p* < 0.000, respectively), and a correlation between CT90% and Shimmer% and HNR values (*p* < 0.016 and *p* < 0.003, respectively). The difference was significant in MPT between the 1st group and 3rd and 4th groups (*p* < 0.03 and *p* < 0.003, respectively). *Conclusions*: Glottic phonation can be affected, especially in patients whose AHI scores are ≥15. Voice quality can decrease as the degree of OSA increases. The increase in CT90% can be associated with the worsening of voice and can be used as a predictor in the evaluation of voice disorders in the future.

## 1. Introduction

Obstructive sleep apnea (OSA) is characterized by episodic narrowing or collapse of the upper respiratory tract and accompanying oxygen desaturation during sleep [1]. The prevalence of OSA in the general population is reported as 2–4% [2]. It is observed in 2% of women and 4% of men. Respiratory standstill during sleep, snoring, and excessive daytime sleepiness are the main symptoms [3]. Polysomnography is the gold standard method in the diagnosis and determination of the degree of OSA. Factors affecting the formation of OSA include upper airway collapse, low arousal threshold, low dilator muscle activity, and respiratory control dysregulation. The activity of the upper airway muscles during breathing prevents collapse. For this protective mechanism to function properly, arousal must not be seen. During sleep, pharyngeal muscles cannot function due to the low arousal threshold in one-third of OSA patients which contributes to the exacerbation of OSA. Low arousal threshold can be predicted by three PSG features, namely, low AHI, high nadir oxygen saturation (SpO2), and large hypopnea fraction of total respiratory events [4]. Permanent respiratory instability may occur in patients with a low arousal threshold. As well as OSA, it was mentioned that a low arousal threshold may also be a pathophysiological factor in patients with asthma. In the coexistence of OSA and asthma, asthma may negatively affect the phenotype of OSA by lowering the arousal threshold [5]. In addition, a lower arousal threshold has been demonstrated in an overlap syndrome, which includes chronic obstructive pulmonary disease and OSA, compared to OSA alone [6].

Apnea hypopnea index (AHI) is the most important criterion used in determining the severity of OSA, while the percentage of the cumulative times in which oxygen saturation is measured below 90% during polysomnography to total sleep time (CT90%) is important in determining the severity of hypoxemia and desaturation, and as CT90% increases, the severity of OSA will also increase [7,8]. Hypoxemia may be even more effective in increasing inflammation. It is known that inflammation occurs in the upper respiratory tract in OSA patients [9]. Inflammation occurring in the upper respiratory tract may affect phonation.

Snoring is the most common symptom of OSA. It is caused by turbulent airflow in the narrowed airway [10]. Structural pathologies occur in the tissues covering the respiratory tract due to abnormal compressed air flows in the upper respiratory tract in patients with OSA, and this increases the severity of OSA over time. Upper respiratory tract structures take over the main role in voice production and work in harmony with the central nervous system. A disorder that may occur in the upper respiratory tract will affect the perceptual and acoustic characteristics of the voice by affecting resonance and articulation [11,12]. The upper respiratory morphology of OSA-diagnosed patients may vary structurally and functionally compared to normal healthy people. These changes affect voice production through resonance [11,13,14].

The human voice can be evaluated by objective, subjective, and perceptual methods. The scales and questionnaires that are self-answered by the patient can be used for subjective evaluation, while acoustic and aerodynamic sound analysis can be used for objective evaluation. Various computer-based voice analysis programs are used for acoustic voice analysis. Among these, the Praat program (version 6.1.03, Boersma & Weenink), which can be downloaded and used free of charge, provides reliable results. The fundamental frequency (f0) (Hz), Jitter%, and Shimmer%, which are the perturbation parameters, and harmonic/noise ratio (HNR) (dB), which is one of the spectral parameters, can be objectively obtained using the Praat program [15].

The maximum phonation time (MPT) from aerodynamic voice analysis and S/Z ratio measurements can be easily made without the need for additional equipment. One of the most recognized and widely used methods in subjective voice evaluation is the voice handicap index [16]. Kılıç et al. modified the voice handicap index and developed the Turkish version of the voice handicap index (VHI-10), which is easier to apply and consists of 10 questions [17].

In previous studies, voice analysis of patients with moderate and severe OSA was generally evaluated, but voice evaluation of patients with mild to severe OSA of varying degrees was not performed. No study includes MPT and S/Z measurements that can be used in the evaluation of glottic pathologies and pulmonary functions, which are aerodynamic methods, in OSA. Similarly, the effects of desaturation and hypoxemia, which affect the weight of OSA, on the voice were not evaluated. We aimed to determine the effect of the degree of OSA on voice and the relationship of CT90%, with the results of the voice analysis, based on the AHI in patients with whom we performed the acoustic analysis using the Praat program, aerodynamic voice analysis evaluating MPT and S/Z ratios, and subjective voice analysis using VHI-10, with the prediction that pathological changes may occur in the upper respiratory tract of OSA patients.

## 2. Materials and Methods

Consent was given by Malatya İnönü University Clinical Research Ethics Committee before the study (2018/91—27 June 2018). Patients between the ages of 18–60 years who were admitted to the otorhinolaryngology or chest diseases outpatient clinics with complaints of snoring, respiratory standstill at night and daytime sleepiness, etc., and were pre-diagnosed with OSA were evaluated. Endoscopic and stroboscopic ENT examination, chest examination, and respiratory function test (Jaeger Vyntus Spiro, Irvine, CA, USA) were performed on all patients. Patients whose examinations and respiratory function test values were normal (FEV1 > 80%, FVC > 80%, FEV1/FVC > 80%) were included in the study. Those who used cigarettes, alcohol, or drugs, had chronic respiratory, had neurological, cardiac, hepatic, or endocrinological diseases, worked in jobs that could be exposed to chemical vapor chronically, or had previously undergone surgery due to any pathology in the respiratory tract were not included in the study. In total, 72 patients who met the criteria were included in the study on a voluntary basis. Patients’ overnight polysomnography was recorded using a 55-channel computerized system (Alice 6 ^®^ Sleepware, Philips Respironics, PA, USA). Polysomnography includes four-channel electroencephalography, two-channel electrooculography, one-channel submental muscle electromyography (EMG), two-channel EMG placed on both anterior tibial muscles, one-channel nasal cannula for oro-nasal airflow measurement, one-channel oro-nasal thermal sensor, two-channel inductive plethysmography to demonstrate respiratory effort in the thorax and abdomen, one-channel “body position” sensor to detect body position, a channel finger probe and a pulse oximeter measuring arterial oxyhemoglobin saturation (SPO2), and simultaneous video recording. The evaluation of sleep stages and respiratory cases observed during sleep was made according to the criteria of the “American Academy of Sleep Medicine (AASM)” [18]. Apnea was defined as the interruption of oro-nasal airflow for at least 10 s. Hypopnea was defined as a 3% decrease in oxygen saturation with at least a 50% decrease in oro-nasal airflow or its accompanying arousal monitoring. Arousal was defined as waking up while sleeping or returning to a more superficial sleep phase. The cases were classified as AHI ≥ 5–15/h (mild), AHI 16–30/hour (moderate), and AHI > 30/h (severe) OSA according to the standard model accepted in the literature. The percentage of the total time (CT90%) during which the oxygen saturation in the blood measured by pulse oximetry was below 90% was recorded during the entire sleep.

### 2.1. Voice Analysis

According to the AHI, volunteers were divided into 4 equal groups: normal (1st group), mild OSA (2nd group), moderate OSA (3rd group), and severe OSA (4th group). Then, the VHI-10 questionnaire was completed. Following, patients were asked to say the vowels /α:/ and /i:/ for 5 s in a voice-isolated audiometry booth. Meanwhile, the voices of the volunteers were recorded on a laptop with the aid of an external-integrated microphone (SAMSON C01UPRO; Samson Technologies, Hauppauge, New York, NY, USA) using the Audacity audio recording program (version 2.1.2, Audacity® software is copyright © 2022–2021 Audacity Team, GNU General Public License). In addition, MPT and S/Z ratios were recorded. For MPT measurement, the patients were asked to make the vowel sound /α:/ for the longest time after a deep inspiration, and the time was recorded. For the S/Z ratio, the patients were asked to separately make the /s/ and /z/ sounds for the longest time they could say after a deep inspiration, and the durations were divided into each other. The five-second recordings obtained from the Audacity program were analyzed using the Praat voice analysis program (Praat version 6.1.03, Boersma & Weenink, GNU General Public License, University of Amsterdam, Amsterdam, The Netherlands) after discarding the one-second parts from the beginning and the end. Jitter%, Shimmer%, HNR, and f0 values were obtained. The results of the acoustic analysis, aerodynamic analysis, and VHI-10 questionnaire were evaluated. Any possible difference between the groups were investigated. Thus, the effects of OSA severity on phonation were sought. The correlation between CT90% and voice analysis results was evaluated. No invasive procedure was performed on the volunteers.

### 2.2. Statistical Method

Statistical evaluation was performed using SPSS (IBM SPSS Statistics 25, Armonk, NY, USA) software. ANOVA statistical analysis and a post-hoc test were applied to understand the difference between the groups. The relationship between CT90% values and voice analysis results was evaluated using the Pearson correlation test. The significance level was taken as *p* < 0.05. Data are reported as mean with range (minimum (min)—maximum (max)).

## 3. Results

The G*power 3.1 program (Hurricane and Typhoon trademarks) was used for power analysis. While the type 1 error (alpha) is 0.05, the power of the test (1-beta) is 0.95, and the effect size is 0.55, the minimum sample size required to find a significant difference using this test should be 64 (16 in each group). Power analysis supported that the number of samples taken was sufficient. Seventy-two patients were included in the study. Each of the four groups consisted of 18 people. The study included 37 females and 35 males. The ages of the patients ranged from 18 to 60 (mean 43) years. The ages of groups ranged from 19–57 (mean 42), 25–54 (mean 41), 26–60 (mean 44), and 18–57 (mean 45) years from the 1st group to the 4th group, respectively. The mean ages of the patients in the groups were similar (*p* > 0.6). The body mass index (BMI) of groups ranged from 26–34 (mean 30.6), 20–40 (mean 30), 25–48 (mean 34.5), and 23–41 (mean 33.8) kg/m^2^ from the 1st group to the 4th group, respectively. The mean BMI of the patients in the groups was similar (*p* > 0.1). Age and weight gain affect voice formation. The fact that the age and BMI values of the groups were similar prevented erroneous results due to these factors. 

### 3.1. Voice Analysis

#### Aerodynamic Voice Analysis

The mean MPT was found to be 20.67 (minimum 9, maximum 27) s, 17.06 (minimum 5, maximum 28) s, 15.44 (minimum 7, maximum 25) s, and 14.06 (minimum 5, maximum 22) s from 1st group to 4th group, respectively. There was a significant difference between the 1st group and 3rd group (*p* < 0.03) and between the 1st group and 4th group (*p* < 0.003).

The mean S/Z values were found to be 0.99 (minimum 0.73, maximum 1.63), 0.90 (minimum 0.58, maximum 1.50), 0.89 (minimum 0.48, maximum 2.20) and 0.92 (minimum 0.55, maximum 1.45) from 1st group to 4th group, respectively. There was no significant difference between the groups (*p* > 0.05).

### 3.2. Acoustic Voice Analysis 

#### 3.2.1. Vowel Sound /α:/

There was no significant difference in f0 values between the groups (*p* > 0.05). There was a significant difference between the 1st group and the 4th group (*p* < 0.001), between the 2nd group and the 4th group (*p* < 0.001), and between the 3rd group and the 4th group (*p* < 0.002) for Jitter% measurements. In terms of Shimmer% values, there was a significant difference between the 1st group and the 3rd group (*p* < 0.032), between the 1st group and the 4th group (*p* < 0.001), between the 2nd group and the 4th group (*p* < 0.002), and between the 3rd group and the 4th group (*p* < 0.007). When HNR measurements were evaluated, there was a significant difference between the 1st group and the 4th group (*p* < 0.001), between the 2nd group and the 4th group (*p* < 0.018), and between the 3rd group and the 4th group (*p* < 0.018) (Table 1).

**Figure 1:**
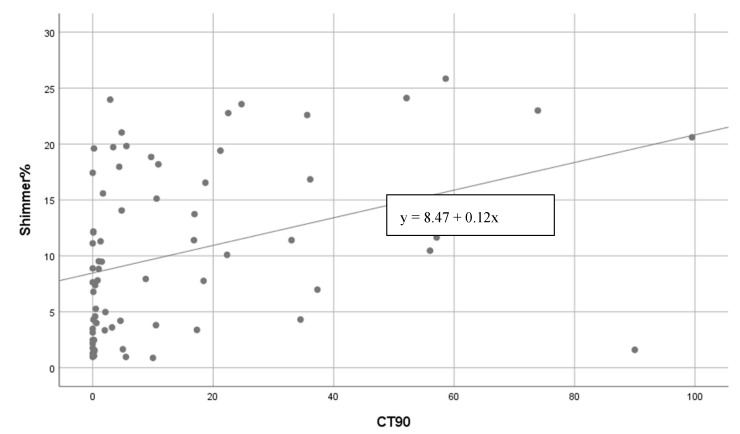

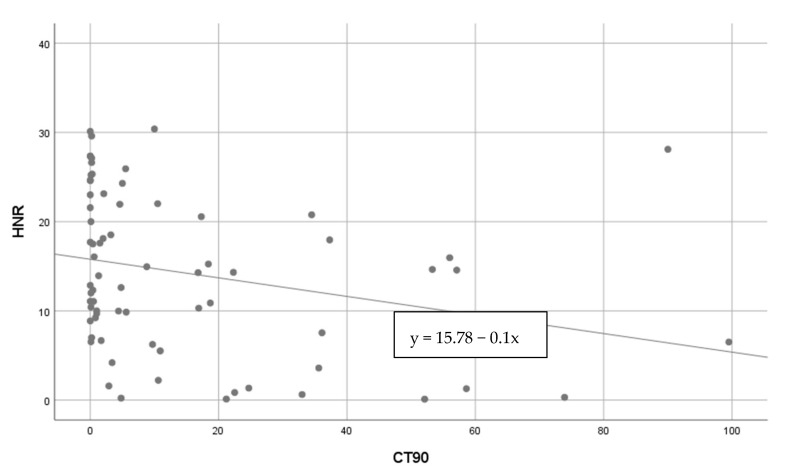
Positive correlation between Shimmer% and CT90%, and negative correlation between HNR (dB) and CT90% (vowel sound /a:/).

The mean CT90% value in all patients was found to be 14.95%. While there was a significant positive correlation between CT90% and Shimmer% values for the vowel sound /α:/ (*p* < 0.001), there was a significant negative correlation between CT90% and HNR values (*p* < 0.021) (Figure 1). There was no significant correlation between Jitter, f0, MPT, S/Z values, and CT90% (*p* > 0.05). 

#### 3.2.2. Vowel Sound /i:/

There was a significant difference in f0 values between the 1st group and the 2nd group and between the 1st group and the 4th group (*p* < 0.028, *p* < 0.015). There was a significant difference between the 1st group and the 4th group (*p* < 0.04) and between the 2nd group and the 4th group (*p* < 0.013) for Jitter% results. When the Shimmer% results were evaluated, there was a significant difference between the 1st group and 3rd group (*p* < 0.041) and between the 1st group and 4th group (*p* < 0.000). In terms of HNR, there was a significant difference between the 1st group and the 2nd group (*p* < 0.032) and between the 1st group and the 4th group (*p* < 0.000) (Table 2).

**Figure 2:**
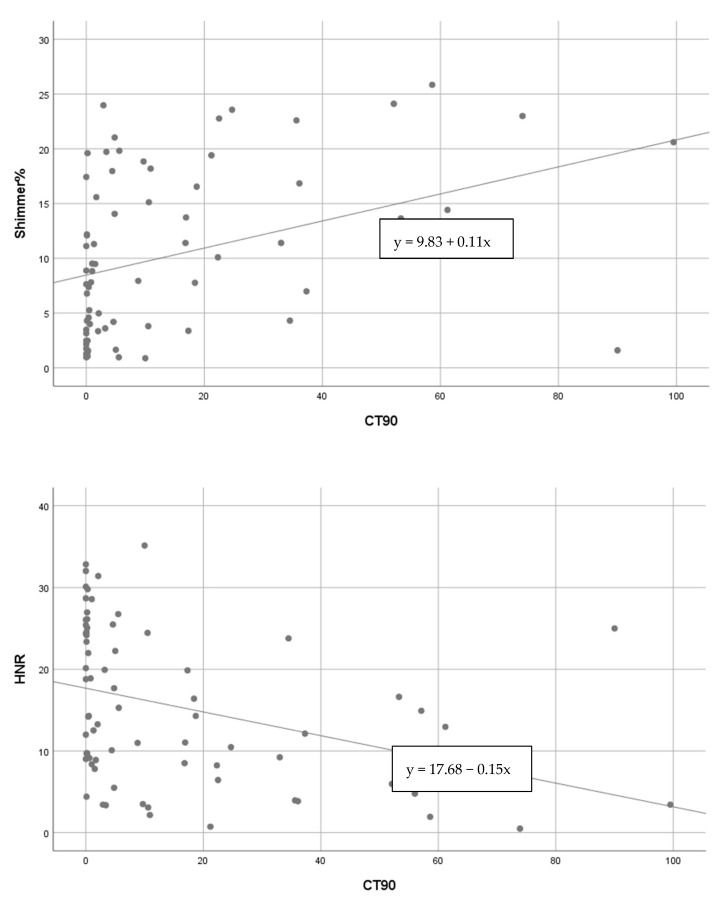
Positive correlation between Shimmer% and CT90%, and negative correlation between HNR (dB) and CT90% (vowel sound /i:/).

While there was a significant positive correlation between CT90% and %Shimmer values in all patients for the vowel sound /i:/ (*p* < 0.016), a significant negative correlation was found between CT90% and HNR values (*p* < 0.003) (Figure 2). There was no significant correlation between Jitter, f0, MPT, S/Z values, and CT90% (*p* > 0.05).

### 3.3. VHI-10 Questionnaire for Subjective Voice Analysis

When VHI-10 was evaluated, the scores of volunteers in the first group were found to be 1 or 0, between 0 and 7 in the 2nd group, between 0 and 16 in the 3rd group, and between 0 and 12 in the 4th group. Although the scores of the other groups were worse compared to the 1st group, no statistical difference was found (*p* > 0.05).

## 4. Discussion

While there are studies on the effect of OSA on voice, especially in patients with severe OSA, there are no studies showing how voice is affected according to the severity of OSA. Furthermore, the correlation between CT90%, which is effective in demonstrating the severity of hypoxemia and desaturation in patients with OSA, and acoustic parameters of voice have not previously been evaluated. We performed acoustic voice analysis using the Praat program, aerodynamic voice analysis by evaluating MPT and S/Z ratios, and subjective voice analysis using VHI-10 in patients with OSA. We evaluated the correlation of the results with CT90%. We found significant differences between the groups in f0, Jitter%, Shimmer%, HNR, and maximum phonation times as the degree of OSA increased according to the voice analysis results. Furthermore, we found a significant correlation between CT90% and perturbation amplitude and HNR. Unlike previous studies, we found significant differences in the voice analyses of mild and moderate OSA patients compared to the normal group. The MPZ of the patients in the moderate OSA group was significantly lower than the normal group, similar to the severe OSA group. Additionally, there was a significant difference between the moderate OSA group and the normal group in Shimmer% values for the vowel sound /a:/. For the vowel sound /i:/, we found a significant difference between the mild OSA group and the normal group in terms of f0 and HNR values. Moreover, Shimmer% values were found to be significantly higher in the moderate OSA group than in the normal group. These results showed that as the severity of OSA, desaturation, and hypoxemia increased in patients, the voice was negatively affected. 

The quality of the voice depends on the regular vibration of the vocal folds and resonance within the vocal tract. The relationship and balance between the opening and closing phases in the vibration of the vocal folds may be impaired due to any pathology. In general, disorders, such as upper respiratory tract inflammation, thickened pharyngeal wall, thickened and sagging soft palate, and hypertrophic tonsils, are commonly observed in patients with OSA, and almost always co-existing snoring is current in the progression of these disorders over time. When the pharyngeal diameter of OSA patients and healthy people was measured while awake and asleep, it was reported that patients with OSA had a significant narrowing in pharyngeal diameter and were more prone to the development of pharyngeal collapse [13]. In a study conducted using acoustic pharyngometry, the minimum cross-sectional areas of the pharynx of people with normal and mild OSA were found to be significantly larger than those of people with moderate and severe OSA [19]. Furthermore, it has been reported that the distance covered by the voice in the upper respiratory tract of patients with OSA increases and that the absorption of the voice may be higher [11]. These pathological changes that may occur in the upper respiratory tract contribute to the formation of abnormal voices by affecting voice production and resonance [14,20,21].

The water (sol layer) that covers the vocal cord epithelium is necessary for vocal cord release and plays an important role in maintaining phonation. This layer may change in cases such as dry air inhalation, breathing through the mouth, snoring, dehydration, or taking drugs that cause mucosal dryness [22,23]. OSA patients breathe completely through the mouth. It has been predicted that with the decrease in moisture in the sol layer, the threshold voltage of vocalization will increase, and the vocal cords may be damaged when the glottic pressure increases to a certain degree [24]. In a study conducted on excised animal larynxes, it was reported that dry air would reduce laryngeal performance by reducing vocal cord hydration [25]. Inflammation and dryness in the upper respiratory tract due to snoring and sleeping with the mouth open may negatively affect the health of the vocal cords and cause dysfunction in phonation. It has been shown that there is a decrease in the voice quality of those who chronically snore compared to those who do not snore [26].

Depending on the change in the mechanical properties of the vocal cords, f0 may vary. When the vocal cord length and subglottic pressure increase and the opening-closing cycle of the cords shortens, f0 increases, while it decreases in the opposite cases. When there is no structural change in the glottic region, the basic frequency is generally not affected. No difference was observed in basic frequency values compared to preoperative values in studies conducted on patients who underwent a wide variety of surgeries that did not include the glottic region [27]. While pharyngeal collapse may be observed in OSA patients, it has been shown by Rubinstein et al. that glottic collapse may also occur [28]. In this study, it was found that the glottic section areas of severe OSA patients without pharyngeal collapse were significantly less than those with only snoring. Different pressures may occur in the respiratory tract of patients with OSA, and basic frequency values may be lower than in healthy individuals [11,29]. Fiz et al. showed differences in laryngeal behavior between OSA and non-OSA volunteers in their study [30]. They examined the harmonic structure in the production of vowels and found that OSA subjects used a narrower frequency range in their production of vowels and had a decrease in the maximum frequency in acoustic analysis. Unlike these studies, Atan et al. found that the f0 values of moderate and severe OSA patients were similar to those of the control group [31]. Furthermore, Wei et al. found the f0 values of severe OSA patients for the vowel sound /i:/ to be similar to the control group [32]. The f0 values for the vowel sound /i:/ of patients with mild and severe OSA were found to be significantly lower than those in the control group in our study. This has shown us that OSA can affect the glottic region and cause pathological changes.

Perturbation parameters are effective in the evaluation of voice quality. Jitter, also called frequency perturbation, is important in evaluating the regularity of vocal cords. As the Jitter% value increases, the voice will become rough and its quality will decrease. The shimmer shows the change in amplitude in each glottic cycle. As the value of Shimmer%, which is known as amplitude perturbation, increases similarly to Jitter%, the voice quality will decrease. In patients with different glottic disorders, there was an improvement in perturbation parameters after voice therapy, in other words, the voice quality of the patients increased [33,34]. Wei et al. stated that the vocal cord vibrations of the patients were irregular in their study conducted on 75 severe OSA patients [32]. They found that there were significant differences in Jitter%, Shimmer%, and noise-harmonic ratio (NHR) values in OSA-diagnosed patients compared to 46 healthy people. These differences applied to measurements they obtained from the vowel sound /i:/ with both MDVP and the Praat program and showed that Shimmer% may be more effective in evaluating the voice of OSA-diagnosed patients. In the study of Karakurt et al. [21], pre-treatment Shimmer% values of patients who were diagnosed with moderate or severe OSA with CPAP-use indication were significantly higher than the control group. Furthermore, NHR values were found to be significantly higher and Jitter% values were found to be similar. Similarly, in the study conducted by Atan et al., Shimmer% values were found to be significantly higher than the control group, although Jitter% values were found to be similar in OSA patients whose AHI scores were >15 [31]. In our study, Jitter% and Shimmer% values of severe OSA patients were found to be significantly higher than all other groups and Shimmer% values of moderate OSA patients were found to be significantly higher than the values of the group with normal diagnosis in the analysis of the vowel sound /a:/. In the evaluation of the vowel sound /i:/, Jitter% values of severe OSA patients were found to be significantly higher than normal and mild OSA patients and Shimmer% values of moderate and severe OSA patients were found to be significantly higher than the normal group. These differences which we found in perturbation parameters showed that there was an irregularity in the vocal cord vibrations, especially of severe and moderate OSA patients.

The increase in HNR indicates that the noise ratio in the voice decreases. An additional noise occurs due to turbulent airflow in the glottis during phonation, indicating the relative amount of additional noise in the HNR voice signal [35,36]. Insufficient closure of the vocal cords causes turbulence by allowing excessive airflow through the glottis. The resulting friction noise is reflected at a higher noise level in the spectrum [37]. Noise in the signal may also be caused by aperiodic vocal cord vibration. Thus, the ratio reflects its dominance over the harmonic (periodic) and noise (non-periodic) levels in the sound and is measured in dB. Perceptually, HNR reflects voice quality. Indeed, HNR has been reported to be an important predictor of perceptually rough voice samples [38,39,40,41]. HNR will decrease in glottic pathologies after long-term voice use [42]. Benavides et al. compared the voice recordings of severe OSA patients with individuals without or with mild OSA using the Praat voice analysis program and found that HNR values were significantly lower in the patients with severe OSA. In the same study, Jitter% values were found to be significantly higher in severe diagnosed-OSA patients, but no significant difference was found in Shimmer% values [43]. Pozo et al. compared 40 patients whose AHI scores were above 30 and 40 patients whose AHI scores were below 10 and found that HNR values were significantly higher in severe OSA patients [44]. While the HNR values were found to be significantly lower in the severe OSA group compared to all other groups for the vowel sound /a:/, it was significantly lower in the mild and severe OSA groups compared to the normal group for the vowel sound /i:/ in our study. This is a symptom of increased noise in the voice and decreased voice quality, especially in patients with severe OSA.

Maximum phonation time and S/Z ratio are used for aerodynamic voice analysis. Especially in glottic pathologies, MPT may be shortened. In the study of Karlsen et al., the MPT results of patients with degenerative/inflammatory laryngeal pathology were found to be longer than the control group [45]. Unlike this study, there was no change in postoperative MPT results in patients who underwent tongue base and pharynx surgery due to OSA. However, no surgery was performed on the glottic region in these studies [46,47]. The S/Z ratio allows evaluation of the degree of glottic closure and pulmonary functions. The S/Z ratio is useful in measuring the adequacy of the laryngeal valve. Normally, these times are expected to be very close to each other. In cases where glottic closure is not complete and resonation is impaired, it is expected that the Z-time will decrease and the S/Z ratio will increase [48,49]. Since the glottic area is affected by glottic pathologies such as vocal cord nodules and masses and following thyroplasty surgeries, changes in S/Z ratios may be observed [50,51]. In this study, the MPT was found to be significantly shorter in the moderate and severe OSA groups. This suggests that glottic pathologies may occur as the severity of OSA increases. We did not find a significant difference in the S/Z ratios.

The voice handicap index is used in the subjective evaluation of voice. As the scores increase, it may be thought that there is a decrease in the voice quality and a disorder in the perceived sound of the person. Wheeler et al. reported that acoustic sound parameters were associated with total VHI scores [52]. Atan et al. reported that 27 severe and moderate OSA patients had worse vocal functions and accompanying worse VHI scores than normal individuals [31]. Similarly, Wei et al. found significantly higher VHI-10 scores in severe OSA patients [32]. Unlike these studies, Hsiung et al. found a low correlation between acoustic voice parameters and VHI [53]. In our study, the VHI-10 scores of the groups were similar and we could not find a correlation between the VHI-10 scores and acoustic voice parameters.

Oxygen desaturation and hypoxemia detected in patients during sleep are among the factors that affect the increase in inflammation. The evaluation of CT90% and AHI will be more effective in determining the severity of OSA [54]. Chaudhary reported that evaluating the ratio of the time spent below 90% oxygen saturation to the whole time would be a more effective method of determining oxygen saturation changes [7]. Similarly, Hoshino et al. stated the importance of CT90% in their study [8]. Inflammation is also expected to increase as CT90% increases. High-sensitivity C-reactive protein (hsCRP) is one of the biological markers of systemic inflammation in OSA patients. Zhang et al. showed that the rate of sleep duration under 90% oxygen saturation was the most important independent risk factor for the increase in hsCRP [55]. In other words, as CT90% increases, hsCRP and inflammation will also increase. Upper respiratory tract inflammation will also negatively affect vocal phonation [26]. While there was a significant positive correlation between CT90% and %Shimmer values for both /a:/ and /i:/ vowel sounds in our study, a significant negative correlation was found between CT90% and HNR values. CT90% should be evaluated as a predictor that voice may be adversely affected in OSA.

There are several limitations of the study. The number of patients could have been higher, and women and men could have been evaluated separately. In addition, data obtained using different sound analysis programs could have been compared. 

## 5. Conclusions

Our findings showed that glottic sound formation was affected, especially in patients whose AHI scores were 15 and above, and that voice quality decreased compared to healthy individuals as the degree of OSA increased. It has also been shown that the increase in CT90% is associated with the worsening of voice parameters and this could be used as a predictor in the future evaluation of voice disorders in OSA. Studies on large populations will be needed to better inform the relationship between CT90% and voice disorders.

## Figures and Tables

**Table 1:** Acoustic analysis results between groups for vowel sound /α:/.

Vowel Sound /α:/	Mean f0 (Hz)		Mean Jitter%		Mean Shimmer%		Mean HNR (dB)	
	Min	Max	Min	Max	Min	Max	Min	Max
1st group	179		0.23 * *^p^* ^≤ 0.00^		4.58 * *^p^* ^≤ 0.00, **** *p* ≤ 0.032^		20.66 * *^p^* ^≤ 0.00^	
	79	308	0.09	0.44	0.97	17.43	8.87	30.11
2nd group	140		0.22 ** *^p^* ^≤ 0.00^		9.35 ** *^p^* ^≤ 0.002^		14.60 ** *^p^* ^≤ 0.018^	
	76	289	0.13	0.39	1.6	20.60	6.51	28.11
3rd group	156		0.86 *** *^p^* ^≤ 0.002^		10.28 *** *^p^* ^≤ 0.007^		14.60 *** *^p^* ^≤ 0.018^	
	76	245	0.16	4.76	0.88	23.97	0.31	30.39
4th group	134		3.24		17.06		7.04	
	81	320	0.26	9.85	10.09	25.84	0.10	15.96

* between 1st and 4th group, ** between 2nd and 4th group, *** between 3rd and 4th group, and **** between 1st and 3rd group.

**Table 2:** Acoustic analysis results between groups for vowel sound /i:/.

Vowel Sound /i:/	Mean f0 (Hz)		Mean Jitter%		Mean Shimmer%		Mean HNR (dB)	
	Min	Max	Min	Max	Min	Max	Min	Max
1 st Group	4.58 * *^p^* ^≤ 0.028,^ *** *^p^* ^≤ 0.015^		0.25 * *^p^* ^≤ 0.04^		5.08 * *^p^* ^≤ 0.00,^ **** *^p^* ^≤ 0.041^		22.76 * *^p^* ^≤ 0.00,^ *** *^p^* ^≤ 0.032^	
	100	322	0.10	0.74	0.60	21.06	8.37	32.82
2 nd Group	137		0.46 ** *^p^* ^≤ 0.013^		11.09		15.07	
	79	296	0.18	1.70	0.79	24.54	3.42	32.03
3 rd Group	161		1.18		12.10		15.61	
	79	275	0.12	5.52	0.91	24.77	0.49	35.12
4 th Group	131		2.08		17.55		8.59	
	86	238	0.11	8.66	6.63	26.40	0.73	17.68

* between 1st and 4th group, ** between 2nd and 4th group, *** 1st and 2nd group, and **** between 1st and 3rd group.

## Data Availability

The datasets are not publicly available but are available from the corresponding author upon reasonable request.
